# Breast cancer circulating tumor cells with mesenchymal features—an unreachable target?

**DOI:** 10.1007/s00018-021-04064-6

**Published:** 2022-01-20

**Authors:** Justyna Topa, Peter Grešner, Anna J. Żaczek, Aleksandra Markiewicz

**Affiliations:** Laboratory of Translational Oncology, Intercollegiate Faculty of Biotechnology, University of Gdańsk and Medical University of Gdańsk, Debinki 1, 80-211 Gdansk, Poland

**Keywords:** Metastasis, Liquid biopsy, Single cell analysis, CTCs enrichment, Positive selection, Negative selection

## Abstract

Circulating tumor cells (CTCs) mediate dissemination of solid tumors and can be an early sign of disease progression. Moreover, they show a great potential in terms of non-invasive, longitudinal monitoring of cancer patients. CTCs have been extensively studied in breast cancer (BC) and were shown to present a significant phenotypic plasticity connected with initiation of epithelial-mesenchymal transition (EMT). Apart from conferring malignant properties, EMT affects CTCs recovery rate, making a significant portion of CTCs from patients’ samples undetected. Wider application of methods and markers designed to isolate and identify mesenchymal CTCs is required to expand our knowledge about the clinical impact of mesenchymal CTCs. Therefore, here we provide a comprehensive review of clinical significance of mesenchymal CTCs in BC together with statistical analysis of previously published data, in which we assessed the suitability of a number of methods/markers used for isolation of CTCs with different EMT phenotypes, both in in vitro spike-in tests with BC cell lines, as well as clinical samples. Results of spiked-in cell lines indicate that, in general, methods not based on epithelial enrichment only, capture mesenchymal CTCs much more efficiently that CellSearch^®^ (golden standard in CTCs detection), but at the same time are not much inferior to Cell Search^®^, though large variation in recovery rates of added cells among the methods is observed. In clinical samples, where additional CTCs detection markers are needed, positive epithelial-based CTCs enrichment was the most efficient in isolating CTCs with mesenchymal features from non-metastatic BC patients. From the marker side, PI3K and VIM were contributing the most to detection of CTCs with mesenchymal features (in comparison to SNAIL) in non-metastatic and metastatic BC patients, respectively. However, additional data are needed for more robust identification of markers for efficient detection of CTCs with mesenchymal features.

**Supplementary Information:**

The online version contains supplementary material available at 10.1007/s00018-021-04064-6.

## Introduction

The process of metastasis is mediated by cancer cells, which entered blood vessels and are transported to distant organs. Detection of such cells, circulating tumor cell (CTCs), in solid tumors, is related to poor prognosis [1, 2] and therapy resistance [3, 4]. As isolation of CTCs is minimally invasive procedure, requiring collection of only a few milliliters of peripheral blood, it shows great potential for monitoring of the disease. Methods of enumeration and profiling of CTCs have been extensively studied, what allowed for the development of Food and Drug Administration (FDA)-approved CellSearch^®^ system [2, 5] and initiation of a large number of clinical trials in which interventions are based on the number of CTCs [5, 6]. However, accurate enumeration of CTCs seems to be more challenging than anticipated, as CTCs show remarkable phenotypic plasticity as a result of the activation of epithelial-to-mesenchymal (EMT) transition process. As a consequence of EMT, CTCs shift their profile of markers expressed from epithelial to mesenchymal, what hinders detection of cells with mesenchymal features [7, 8]. EMT enhances migratory potential, survival and stem cell phenotype of cancer cells—features involved in successful metastatic progression [9–11]. Validation and translation of these finding in patients samples is crucial, as it will justify shifting the focus to CTCs with mesenchymal features. Indeed, progression of the disease was linked with increase of mesenchymal CTCs fraction [3, 12], indicating that reliable assessment of CTCs burden requires quantification of both epithelial and mesenchymal CTCs subpopulations. A number of methods for CTCs isolations from breast cancer (BC) patients have been described in extensive review articles [13–15], however potential of these methods (group of methods) in isolating CTCs of different EMT status was not systematically compared. What constitute a good mesenchymal marker for CTCs detection is still a matter of debate, especially considering that a number of proteins were found to induce EMT or be upregulated during EMT in BC, but exact mechanism of their involvement in EMT is not well examined. Well-known EMT effectors, like vimentin (VIM) [9, 16], N-cadherin [9, 16], or core regulators—EMT transcription factors (EMT-TFs) TWIST1 [17–19], SNAIL [17–20] SLUG [17, 19], ZEB1 [17–19] are frequently considered as markers of EMT. But apart from these well-established markers, other proteins were found to be increased in EMT-undergoing cancer cells or were involved in induction/maintenance of mesenchymal features of cancer cells; these include: EGFR [21, 22], MCAM [23, 24], MUC1 [25], PI3K [26–28], AKT2 [28, 29], BIRC5 [28, 30], HER2 [31], HER3 [32], TG2 [33, 34], FOXC2 [35, 36], MAGEA3 [37], AURKA [38, 39], MGB1 [40], PLS-3 [41]. Therefore in the current work, to define mesenchymal trait, we included a wider range of markers, which are non-epithelial in origin, but which were found to be related to mesenchymal features of cancer cells. In this respect, these markers can be found in cells with different mesenchymality along the EMT spectrum, including cells with intermediate epithelial-mesenchymal features.

We described and compared selected methods which were designed to isolate epithelial, as well as mesenchymal CTCs, and compared their recovery rates (RRs) from spike–in experiments with BC cell lines of varying EMT status. Nevertheless, from all of the developed methods only limited number was investigated on BC patients samples and showed their ability to capture mesenchymal CTCs. Additionally, we summarized data from clinical studies of BC patients, which gave positivity rates (PRs) of CTCs identified by different markers related to mesenchymal phenotype, then we employed statistical modeling to establish which CTCs enrichment method and markers are characterized by the lowest/highest CTCs PR, what can help to guide future basic and translational research in the field of BC liquid biopsy.

## Clinical significance of mesenchymal CTCs

### Prognostic significance

Mesenchymal phenotype of CTCs has been detected in BC patients within the wide range of 0–100%, depending on the markers used for detection and stage of the disease. Several studies have shown improved CTCs detection by employing EMT markers (such as VIM, cell-surface VIM (csVIM), MCAM, EMT TFs—TWIST1, SNAIL, SLUG, ZEB1, FOXC2) in addition to epithelial markers [12, 42–45]. Presence of mesenchymal markers (VIM and TWIST1) was higher in metastatic BC patients than in early-stage BC patients [44, 46, 47] and detection of mesenchymal CTCs (CK19^−^/VIM^+^) was associated with lymph node involvement [48], suggesting that EMT phenotype is directly related to the metastatic potential of CTCs. Additionally, detection of mesenchymal CTCs (with expression of VIM) correlated with poor patients outcome both in early [49–51] and metastatic BC (CTCs negative for epithelial cytokeratins, but expressing VIM or fibronectin) [52]. Metastatic breast cancer (MBC) patients with high expression of EMT-TFs TWIST1, SNAIL and SLUG undergoing high-dose chemotherapy followed by autologous hematopoietic stem cell transplantation had shorter progression-free survival (PFS) compared to patients with low expression of these EMT-TFs [53]. Furthermore, in the study of 594 BC patients of all stages, mesenchymal CTCs (plastin 3-positive) were shown to be an independent prognostic marker of overall survival (OS) and disease-free survival (DFS) in multivariate analysis [41]. Detection of CTCs using csVIM enrichment and CD45 depletion (allowing to isolate mesenchymal CTCs) was shown to identify patients with progressive disease more accurately than CellSearch^®^, which primarily captures CTCs with epithelial features [12]. Concordance rate between the two methods was 66.67% in patients with stable disease but decreased to 45% in case of patients with progressive disease, indicating the role of EMT in BC progression.

CTCs with EMT and stemness features (characterized by *TWIST1* and *ALDH1* expression, respectively) correlated with lung metastases and decreased PFS in MBC patients [54]. Similarly, presence of mesenchymal markers (*VIM* and *TWIST1* transcripts) in CTCs isolated with CanPatrol™ correlated with shorter PFS in MBC patients enrolled to prospective phase III CAMELLIA study [55]. The authors concluded that the criteria, which combine the total CTCs count and the proportion of mesenchymal CTCs may be used to monitor therapeutic resistance and predict prognosis in MBC [55]. Also, in operable BC (stage I–III) detection of mesenchymal CTCs correlated with significantly shortened OS [50, 51] and DFS [56]. Importantly, mesenchymal CTCs allowed more accurate prediction of prognosis than the expression of epithelial markers alone [52]. The proportion of mesenchymal CTCs appeared to be more accurate in predicting disease progression than total CTCs count and serum tumor markers level (CA153, CEA), and was able to identify it earlier than radiographic examination [55].

Literature data argue that cells with intermediate EMT phenotype, and not necessarily the ones that completed EMT, might be the most aggressive. Recently, it was demonstrated in vivo that tumor cells proceed through various hybrid epithelial/mesenchymal states with differing invasive, metastatic, and differentiation characteristics [57]. Carcinoma cells residing in intermediate epithelial-mesenchymal phenotypic state, rather than a mixture of cells with either epithelial or mesenchymal phenotype, appear critical to successful metastasis [58]. Armstrong et al. showed that the majority (> 80%) of CTCs co-expressed epithelial and mesenchymal proteins in patients with progressive metastatic solid tumors, including castration-resistant prostate cancer and MBC [59]. Similarly, in a large cohort of Chinese BC patients (*n* = 1083) more than 73% presented epithelial-mesenchymal phenotype of CTCs based on the expression of *EpCAM, CK8, CK18* and *CK19* as epithelial, and *VIM* and *TWIST1* as mesenchymal markers [44]. Both mesenchymal and dual epithelial-mesenchymal phenotype were associated with distant metastases [44]. MBC patients with CTCs co-expressing epithelial and mesenchymal markers had shorter PFS and OS [60], while no prognostic impact of intermediate phenotype in operable BC was observed [51].

Mesenchymal CTCs were also shown to circulate in blood as clusters, and lately, clinical significance of such clusters has gained significant attention [3, 61]. CTCs from BC patients were shown to contact each other within aggregates via cytoskeleton bridges supported by VIM, α-tubulin, and detyrosinated α-tubulin [62]. In MBC presence of CTCs clusters was associated with worse clinical outcome as compared with the presence of single CTCs [61, 63–65]. CTCs clusters were identified in 16 out of 54 CTCs-positive MBC patients (29.6%) [61]. Patients with triple-negative and HER2-positive BC had CTCs clusters more frequently than patients with hormone receptor-positive cancer [65]. Patients with detectable clustered CTCs, particularly with the continuous presence of clusters across different time points, had significantly shorter PFS than those with single CTCs [61]. Another prospective study confirmed prognostic significance of CTCs clusters showing that the presence of CTCs clusters was a strong independent predictor of PFS in advanced-stage BC patients, particularly in patients with inflammatory BC [64]. Importantly, CTCs clusters added prognostic value to CTCs enumeration alone, and a larger-size CTC-clusters conferred a higher risk of death in MBC patients [66].

### Predictive significance

Although increasing data and clinical trials show that CTCs can improve prognostic accuracy both in early and metastatic settings, their predictive role is less extensively reported, particularly scarce are data concerning predictive role of mesenchymal CTCs. The majority of reports on the possibility of monitoring therapeutic efficacy concern enumeration of CTCs, based on EpCAM with the use of CellSearch^®^ system [1, 2, 67, 68]. No prospective clinical trials regarding the role of different phenotypes of CTCs were reported. Presence of mesenchymal CTCs (csVIM-positive) was associated with progressive disease in BC patients undergoing post-surgery adjuvant chemotherapy [69]. MBC patients not responding to therapy showed increased level of EMT markers (assessed by TWIST1, PI3Kα, AKT2) compared to responders [70]. First-line chemotherapy in MBC patients resulted in significant increase in the incidence of cancer stem cell^+^/partial EMT^+^ (ALDH1^+^/TWIST1^+^) CTCs, suggesting that those CTCs represent a chemoresistant subpopulation [54]. In primary BC patients CTCs expressing EMT-TFs occurred more frequently in neoadjuvant treated subgroup, what inspired authors to conclude that neoadjuvant treatment was not able to eliminate CTCs with EMT phenotype [7]. EMT status of CTCs was recently investigated in eribulin-treated MBC patients [71]. It was reported that total CTCs at baseline, comprised of mesenchymal CTCs (VIM^+^) and epithelial CTCs, might predict eribulin efficacy. Total CTCs number was significantly increased in the group of patients with progressive disease, with mesenchymal CTCs showing a similar tendency. The expected decrease in mesenchymal CTCs count in responders to eribulin was not observed. However patients with more mesenchymal CTCs at baseline had significantly shorter PFS [71].

Importantly, CTCs phenotype variation during the course of treatment may serve as pharmacodynamic monitoring tool. In non-responding MBC patients number of mesenchymal CTCs (fibronectin 1/N-cadherin/SERPINE1 detected by RNA in situ hybridization) increased, while responders presented decrease in CTCs number and/or proportional decrease in mesenchymal (compared to epithelial) CTCs [3]. These findings remain in agreement with studies highlighting the importance of EMT in conferring chemoresistance in breast and pancreatic cancer models [72, 73]. Recently, short-term expansion of CTCs in microwell-based culture was reported to predict response to anti-cancer therapy in early stage, locally advanced and MBC [74]. Cluster formation in cell culture was affected by the presence and duration of systemic therapy, and cluster persistence may reflect therapeutic resistance as it correlated with shorter OS [74]. Also gene expression profiles in CTCs were reported to predict response to therapy with aromatase inhibitors [75] and during the course of palliative treatment in MBC [76].

To summarize, clinical data show that isolation of CTCs with different EMT status and accurate evaluation of the extend of the mesenchymal phenotype in CTCs might bring important information for the assessment of the disease or treatment course. Presence of mesenchymal markers in CTCs was related to the metastatic potential of CTCs in clinical setting. CTCs with mesenchymal phenotype were found to identify patients with progressive disease more accurately than CTCs detected with epithelial marker only [3, 12, 52, 71]. Unfortunately, we are still lacking prospective clinical trials on the role of different EMT phenotypes of CTCs in clinical decision making. Such trials are needed to justify EMT phenotyping or using methods which capture also CTCs with more mesenchymal features. Whereas the analysis of epithelial CTCs with CellSearch^®^ has documented clinical value, less is known about the ability of the current methods to detect mesenchymal CTCs in BC. Next sections describe the current methods known to be able to detect mesenchymal CTCs in model systems (cancer cell lines spiked to blood), as well as in cancer patients. They also aimed at quantitative comparison of mesenchymal CTCs isolation methods and mesenchymal CTCs detection markers to reveal their contribution to effective CTCs isolation strategies.

## Methods for mesenchymal CTCs isolation

### Negative selection

Negative selection is a frequent initial step of many marker-dependent CTCs detection methods [7, 12, 51, 61, 77] (Supplementary Table 1). Most of the CTCs isolation methods using negative selection approach employ magnetic nanoparticles coated with antibodies against common hematopoietic cells marker—CD45. Some use additional markers specific for a given cells subpopulation, like granulocytes marker CD66b, CD15 or endothelial cells marker CD34, what decreases the probability of blood or endothelial cells misidentification as CTCs and increase method specificity [42, 78, 79]. Negative selection increases throughput, but limits sample purity, because not all blood cells express used marker at sufficiently high level to be effectively depleted [48, 80]. Moreover, CTCs may be lost due to their capture between concentrated blood cells moving towards the magnet [81].

#### CD45-based depletion

There are many commercially available kits for blood cells negative selection based on CD45-positive cells depletion. Most common are EasySep™ Human Whole Blood CD45 Depletion Kit and MACS (Magnetic Cell Separation System), which deplete CD45-positive cells by passing cell suspension through a magnet [12, 77, 82], whereas Dynabeads™ CD45 and Dynal-anti-CD45 beads (CELLection™ beads coated with anti-CD45 monoclonal antibody) require incubation of cell suspension on a magnetic stand [48, 50, 51, 83]. The RR of cancer cells using different kits for CD45-positive cells depletion may differ—Kallergi et al. showed that CD45 Miltenyi-anti-CD45 beads (used in MACS) show decreased recovery of spiked cells in comparison to Dynal anti-CD45 beads (19.0 vs 97.3 for epithelial, 33.5 vs 91.5 for mesenchymal BC cell lines) [83].

#### Multimarker-based depletion

The efficiency of negative selection may also be enhanced by employing more than one marker. Such approach was applied in MINDEC (Multi-marker Immunomagnetic Negative Depletion Enrichment of CTCs), which enables CTCs separation from whole blood or PBMCs fraction. This technique employs a panel of biotynylated antibodies directed to pan-leukocyte marker (anti-CD45), and lineage-specific markers, like NK cells and neutrophils (CD16), B-cells (CD19), monocytes and macrophages (CD163), as well as erythrocytes (CD235a). Cells positive for these proteins are depleted from the tested sample with the use of streptavidin-coated magnetic beads (Depletion MyOne™ SA Dynabeads) during incubation on a magnet. MINDEC has been used for EpCAM^−^/MCAM^+^/CD45^−^ single CTCs and CTCs cluster detection in samples obtained from pancreatic cancer patients [84, 85] and allowed for transcriptomic profiling of single cells [85].

Another platforms enabling rare cancer cells isolation from whole blood by negative selection are inertial focusing-enhanced microfluidic CTC capture devices, termed ^neg^CTC-Chips. As sorted cells are not fixed during the procedure, further molecular characterization on RNA level can be performed. Depletion procedure on ^neg^CTCs-Chip consists of (1) separation of erythrocytes and platelets from the nucleated cells by hydrodynamic cells sorting, (2) inertial focusing of nucleated cells and (3) magnetophoretic removal of blood cells coupled with immunomagnetic beads against blood cells, like CD45, CD15, CD66b, CD14 and CD16 [61, 78, 79]. ^neg^CTCs-iChip was applied for single CTCs and CTCs clusters detection in BC patients, and it was possible to detect cells expressing EpCAM, HER2 or CDH11 after depletion of CD45-,CD14 and CD16-positive blood cells [61].

### Marker-dependent positive selection

Ability to isolate cancer cells in pre- and post-EMT states using positive selection approach is still in the development/discovery phase. Due to the lack of appropriate mesenchymal markers, majority of methods isolating CTCs by positive selection rely on expression of epithelial cell surface markers. Since EMT-undergoing CTCs loose expression of epithelial markers, like EpCAM or E-cadherin, they cannot be considered as a golden standard for CTCs isolation with advanced EMT phenotypes. Novel markers and methods are urgently needed to allow the capture of the wide spectrum of CTCs phenotypes. Below we describe methods which used mesenchymal markers (or a combination of epithelial and mesenchymal markers) for isolation of BC cells either from clinical samples or spike-in BC CTCs models.

#### Immunomagnetic methods

The only FDA approved method for CTCs isolation is CellSearch^®^, which captures CTCs with immunomagnetic beads coated with anti-EpCAM antibodies, followed by immunofluorescent staining for CK8, -18, -19, CD45 and DAPI. Some EMT traits can still be detected in cells captured by CellSearch^®^ (described in the Sect. 4), however EpCAM-negative CTCs, like those which have lost their expression through EMT, cannot be detected [7, 8]. One of the CellSearch^®^ modifications, which could allow capturing of more mesenchymal CTCs, includes additional anti-MCAM antibody for CTCs immunomagnetic isolation. MCAM (CD146) is frequently expressed in BC cell lines lacking EpCAM [42], and as a marker for CTCs enrichment may improve detection of the aggressive, EpCAM-negative CTCs population from BC patients [43].

##### AdnaTest BreastCancer

AdnaTest BreastCancer is based on positive selection with magnetic beads coated with antibodies against EpCAM and MUC1, detecting normal and underglycosylated MUC1 epitope, which is more frequently observed in cancer than in hematopoietic cells. The identity of such isolated putative CTCs is further confirmed by quantitative microcapillary electrophoresis of *EpCAM* (*GA733-2)*, *HER2* and *MUC1* transcripts [76, 86, 87]. CTCs detected with AdnaTest EMT-1/Stem Cell Detect in BC patients manifested EMT (*PI3Kα, AKT2, TWIST1*) and stem cell-like features (*ALDH1,* markers additionally analyzed by researchers: *Bmi1, CD44*), what indicated that AdnaTest is suitable for detecting one of most aggressive CTCs in circulation [86].

##### HER2-coated magnetic particles

HER2 has been shown to be a useful marker for CTCs detection [51], but may be also used for its isolation [88]. Anti-HER2 antibodies conjugated with magnetic iron oxide nanoparticles enable isolation of HER2 overexpressing cells, what is especially important as one-fourth of breast cancers overexpress HER2 [89] and HER2-positive CTCs are observed also in patients with HER2-negative tumors [90, 91].

##### rVAR2-binding method

Cancer cells can present on their surface uniquely modified form of glycosaminoglycan—oncofetal chondroitin sulfate (ofCS)—which is composed of proteoglycans with long chains of repeated disaccharides [92, 93]. Normally restricted to the placenta, ofCS can be overexpressed by primary and metastatic tumors, making ofCS a specific cancer biomarker [94, 95]. Salanti et al. described that ofCS can be detected by glycosaminoglycan-binding malaria protein—VAR2CSA [92]. Its recombinant version—rVAR2, binds specifically to various cancer cell lines, including BC cell lines with different EMT status [92, 94, 96]. Since ofCS expression is maintained in cells undergoing EMT and its reverse process—mesenchymal-epithelial transition (MET), rVAR2 binding is suitable for detection of both epithelial and mesenchymal BC cells [94]. The ability of CTCs detection using rVAR2 protein conjugated with magnetic beads was confirmed in blood samples from 44 patients with different types of epithelial cancers. Additionally, no false-positive signals were detected in blood from healthy donors [94], what makes it a promising marker for mesenchymal CTCs detection.

#### Immunomicrofluidic methods

##### LiquidBiopsy^®^

Using LiquidBiopsy^®^ platform CTCs are first labelled with streptavidin-covered magnetic beads (iMAG™, BD Bioscience) connected with biotynylated antibodies directed to surface BC-related proteins, like EpCAM, HER2, Muc-1 or Trop2 [97, 98]. Next, such labelled cells (potential CTCs) are separated from other cells on high throughput sheath flow microfluidics device, which captures cells labelled with magnetic beads.

The platform allows any biotynylated antibody to be used for detection of target cells. Moreover, the bulk of detected cells can be further isolated for downstream analysis and characterization with methods that are compatible with fixed cells [97]. The application of multimarker-based LiquidBiopsy^®^ is superior to employment of EpCAM alone—the median number of CTCs recovered from 32 metastatic BC patients was almost 3 times higher when CTCs were recovered with the set of antibodies cocktail (EpCAM, HER2, Trop2) vs anti-EpCAM antibody alone (23.5 vs 8.0, respectively) [98].

##### NP^HB^CTC-Chip

NP^HB^CTC-Chip is one of the modifications of microfluidic devices enabling CTCs isolation by inertial focusing. Utilization of gold nanoparticles on a herringbone chip enhanced capture efficiency and recovery of isolated CTCs compared to the unmodified ^HB^CTC-Chip platform. Nanoparticles were coated with antibodies against EpCAM, HER2 and EGFR to ensure efficient isolation of cells lacking epithelial surface markers. A validation study on MBC patients showed that NP^HB^CTC-Chip enables detection of EpCAM and/or CDH11-positive single CTCs and CTCs clusters [99].

##### GEDI

GEDI (Geometrically Enhanced Differential Immunocapture) is a microfluidic platform based on immunocapture using chip with the anti-HER2 antibody-coated microposts. Due to specially designed geometry, HER2-positive CTCs may be captured with reduced leukocytes contamination [100]. CTCs (defined as DAPI^+^/cytokeratin^+^/CD45^−^ cells) were successfully isolated from all (*n* = 5) HER2-positive and HER2-negative BC patients; captured cells showed wide range of HER2 expression [101].

##### OncoCEE™

OncoCEE™ microchannels were designed to avoid laminar flow through the chamber, what maximizes cells contact with antibodies-coated microposts’ inner surfaces [102]. The device employs a number of antibodies directed to epithelial and BC markers (EpCAM, HER2, MUC1, EGFR, folate binding receptor, Trop2), as well as mesenchymal and stemness markers (c-MET, N-Cadherin, CD318, mesenchymal stem cell antigen) [103]. The preparation of CTCs with OncoCEE™ platform may require initial enrichment, e.g., by density gradient centrifugation, but allows not only for CTCs capture and visualization, as cells may be intended for downstream characterization with fluorescent in situ hybridization (FISH) directly on the chip [102, 104].

#### Functional methods

##### TelomeScan

One of the hallmarks of cancer cells is unlimited proliferative potential resulting from telomerase activity (reviewed in [105]). In most human cancers, telomerase is expressed at high level, while in normal somatic cells telomerase is repressed [106]. Kojima et al. established a method of CTCs labeling involving telomerase-specific replication-selective adenovirus expressing *gfp* (green fluorescent protein) gene (called OBP-401 or TelomeScan), which enters cells via coxsackievirus-adenovirus receptor (CAR) on cells surface [107, 108]. The modified virus contains human telomerase (hTERT) promoter-driven viral genes for replication of the virus in telomerase-expressing cells and cytomegalovirus-promoter driven *gfp* expression for monitoring viral replication [109]. The method requires preliminary red blood cells lysis, but does not involve additional enrichment step. After sample incubation for 24 h with the virus particles, GFP-positive cells can be visualized, enumerated and subjected for further analyzes [107, 109]. TelomeScan was used for CTCs detection from different types of epithelial cancers, including BC [107, 109–115]. In early and metastatic BC patients TelomeScan showed similar CTCs detection rate to CellSearch^®^, but with little overlap between the two methods [109].

Despite high sensitivity, the main problem of TelomeScan method is the possibility of false-positive results, as normal blood cells, especially monocytes, may become infected and express low levels of GFP [109, 112, 116]. Moreover, CAR receptor used for the virus entry into the cell, is downregulated during EMT and metastatic progression [117, 118]. This problem was solved in improved version of the recombinant virus (OBP-1101, also called TelomeScan F35), where miR 142-3p (ubiquitously expressed in blood cells) regulatory sequences were inserted, allowing for post-transcriptional silencing of the viral and *gfp* genes in blood cells. Besides reduction of non-specific GFP expression, TelomeScan F35 is able to infect also post-EMT and stem cell-like cancer cells, as the modified virus uses different receptor for viral entry—CD46, which is ubiquitously expressed gene [108, 119]. So far TelomeScan F35 was usefully used for CTCs detection in non-small cell lung (NSCL) and cervical cancer patients, showing a lower percentage of false-positive cells [108, 120]. In another study, mesenchymal CTCs detected from NSCL cancer patients were associated with poor drug response and shorter PFS [121]. TelomeScan is superior to classical telomerase staining as detected living cells may be further analyzed on trancriptomic or genomic level.

##### NanoFlares

The NanoFlares technology allows for staining of intracellular markers in living cells, what is often a severe limitation when working with mesenchymal markers. NanoFlares are oligonucleotides-modified nanoparticles taken up via caveolin-mediated endocytosis [122–124]; they are resistant to nuclease degradation, have no cytotoxic effect on cells, thus this method is suitable for living cells detection and their transcriptomic characterization. NanoFlares technique is based on the recognition of mRNAs of target genes by corresponding oligonucleotides attached to gold nanoparticles; in this case gold acts as fluorescent quencher of fluorophore-labelled reporter sequence bound to oligonucleotides. If mRNAs are present in a cell, they bind oligonucleotides on gold particles, replacing fluorophore-labelled reporter sequence, which becomes fluorescent when separated from the gold nanoparticle [125]. To date, NanoFlares have been useful in transcripts detection in many cell lines and primary cells [122, 125]. Application of NanoFlares for CTCs detection in blood sample requires prior erythrocytes and CD45-positive cells depletion. VIM-targeting NanoFlares have also been successfully used in CTCs detection in a xenograft mouse model of human BC [125]. This method can be potentially used for CTCs isolation from BC, but additional CD45-targeting NanoFlares should be used to exclude VIM-expressing blood cells.

### Physical methods

#### Membrane filtration

Filtration relies on larger diameter of CTCs in comparison to leukocytes. The difference in size of cancer and blood cells was extensively reviewed [126] and provides a solid foundation to employ morphology-based CTCs isolation techniques [126–129]. Such platforms rely on filtration through the single-layer microporous membrane (pores diameter 6–9 μm) made with polycarbonate or other synthetic material. Membrane microfiltration enables precise separation of CTCs larger in size, nevertheless does not provide isolation of smaller cancer cells, which can pass through the pores [126]. Unfortunately, some of the platforms require sample enrichment and cells fixation, what prolongs procedure and limits further transcriptomic analysis [127].

##### ISET

ISET (isolation by size of epithelial tumor cells) is one of the most frequently used platform for size-dependent CTCs isolation and is based on filtration through polycarbonate membrane with cylindrical 8 μm pores under vacuum [127]. This technique is easy to perform, rapid, do not cause cell damage or changes in morphology and is suitable for application to a broad range of carcinomas [127, 130, 131].

##### ScreeCell^®^

ScreenCell^®^ device enables isolation of CTCs by filtration of whole blood (fixed or non-fixed) through a polycarbonate membrane with 6.5–7.5 µm in diameter [128]. This technology is characterized by short time of sample processing, which is crucial for viable cells isolation [128, 133] and has been used for CTCs isolation from breast, prostate, renal and lung cancer [134–139]. Downstream analyses, such as staining or FISH, may be performed directly on the filter, or cells can be washed out for further culture or used for another assays [128].

##### Canpatrol™ CTC assay

One of the filtration-based platforms which has been shown to be useful in detection of CTCs with a wide spectrum of EMT phenotypes from BC patients is Canpatrol™ CTC assay. This method is based on erythrocytes lysis, followed by filtration by size through the membrane with 8 µm pores and RNA in situ hybridization (RNA-ISH) for characterization of captured cells. Zhang et al. using Canpatrol™ showed presence of epithelial (*EpCAM*^+^*/CK8*^+^/*CK18*^+^/*CK19*^+^), mesenchymal (*TWIST* and *VIM*) and biphenotypic (epithelial-mesenchymal) CTCs in both early and MBC patients [44].

#### Microfluidic filtration

The employment of microfluidic devices allowed to overcome the difficulties related to filter clogging caused by cells accumulation on the filter during ordinary filtration. Achieving good efficiency and recovery, as well as cells viability may be also hampered by increasing fluid-driven pressure and prolonged adhesion to the filter [140]. The fact that blood sample does not have to be enriched and fixed before processing is a great advantage of microfluidic separation, as faster procedure increases the possibility of obtaining good quality material after whole processing [141–143].

##### Parsortix™

Parsortix™ is size- and deformability-based system enabling CTCs isolation through blood flow within a cassette with a cross-sectional gap (4.5–10.0 μm) under controlled and constant pressure [140, 144]. Larger and less deformable CTCs retain in channels, while blood cells pass through the device. Beside of CTCs enrichment, Parsortix™ allows CTCs detection by staining performed in the cassette. Moreover, viable single cancer cells may be harvested after whole procedure and used for further analyses [145]. Recently, FDA has accepted submission of Parsortix™ system for use with MBC patients.

##### VyCAP

VyCAP involves passing of leukocyte-depleted blood fraction on the microwell chip under a 10 mbar negative pressure [141–143]. Bottom of each of the 6400 microwells on the chip is 70 µm in diameter, transparent silicon nitride membranes with a single 5 µm pore in the centre. Hydrodynamic forces drag individual cells into microwells towards the pore. Because the diameter of the pore is smaller than the cell (potential CTC), when a cell falls onto the pore, the sample flow through that particular microwell stops and no other cell can enter the same microwell; the next cell is diverted to a neighboring well [141, 143]. VyCAP technology requires additional immunofluorescent staining and imaging, what is possible through the transparent membrane on the bottom of the wells [141]. Living cells may be isolated by 50 µm sharp punch needle, pushing the cell into the well of the PCR plate, what allows for transcriptomic, genomic or even proteomic analysis by DNA-based antibody barcoding, as shown in lung cancer patients [146]. However, analysis on clinical samples showed that in MBC, VyCAP captured only up to 5% of CTCs number detected by CellSearch^®^ in parallel experiment [147]. Even if initial depletion of hematopoietic cells may decrease the RR, avoidance of this step can reduce the throughput of VyCAP method, as only 6400 wells are available on single microchip.

#### Inertial microfluidic separation

The devices exploiting inertial microfluidic separation may be based on the spiral channels with a rectangular or trapezoidal cross-section ended with two outlets [148–150]. Importantly, such devices do not require any external forces for cells separation, but rely on fluidic forces cell size. Smaller blood cells affected by Dean forces migrate in the channel along counter-rotating vortices between the inner and outer wall. Larger cancer cells are additionally affected by inertial lift forces, what results in their focusing near the inner wall of the channel and prevents from migrating further under the influence of Dean drag forces [151]. Such application allows continuous rapid separation of viable CTCs and blood cells, with the latter ones depleted as waste from the outer outlet [148, 150].

The clinical utility of a spiral microfluidic devices was confirmed in MBC patients, in all of which CTCs were detected [149, 150]. To the date, the only one commercially available spiral microfluidic device is ClearCell^®^ FX System [152].

Another inertial-focusing-based technology, commercially available as VTX-1 Liquid Biopsy System, is also based on laminar vortices and inertial microfluidics. In contrast to spiral devices, chip consists of 16 parallel channels and serial reservoirs in each channel [153, 154].

#### Dielectrophoresis-based selection

Cancer cells detached from primary tumor and intravasated into circulation differ from cells physiologically present in blood in terms of their phenotype or morphology. Such features determine specific electrical characteristics of CTCs, what may act as attributes to distinguish them from blood cells by dielectrophoresis (DEP) [155–159]. However, if cells are not fixed, changes in permeability of cell membranes might take place, which hampers distinction of true cancer cells [155].

##### ApoStream^®^

DEP-based methods distinguishes CTCs from blood cells based on differences in conductivity which are dictated by cells biophysical characteristics. CTCs are separated using DEP technology in a microfluidic flow chamber, where cancer cells are attracted by positive DEP forces toward the electrode plane, whereas a bulk of blood cells is levitated by negative DEP into the hydrodynamic flow velocity profile [160, 161]. ApoStream^®^ is considered as a DEP-based device with one of the greatest throughput compared to other DEP-based microfluidic chips [155, 160, 162], but device with interdigitated comb-like electrodes showed higher isolation efficiency in spike-in test with mesenchymal BC cell line MDA-MB-231 [160, 161]. ApoStream^®^ enables CTCs capture from many tumors, including BC [163] and is suitable for monitoring changes in CTCs profile during treatment [164].

#### Acoustophoresis-based selection

Acoustic-based approaches for CTCs isolation are basing on differences in size and physical properties of cancer and blood cells, which migrate with different speed under the influence of acoustic radiation forces [165, 166]. Such separation is achieved by a standing acoustic field inside a flow channel [165]. Whereas most of the of the acoustic-based devices are characterized by parallel fluid flow direction to the standing acoustic wave direction [167], approach using tilted-angle standing surface acoustic wave (taSSAW) allows fluid flow at specific angle, improving efficacy and sensitivity of separation [166]. An important advantages of acoustic-based separation are lack of influence on isolated cells genotype and phenotype, as well as good viability after whole procedure [166, 168, 169], what potentially allows further comprehensive analysis of isolated CTCs. The two-stage acoustophoresis chip described by Antfolk et al. also allows simultaneous separation and volume concentration of the sample with isolated cells [170], what is suitable for CTC isolation from cancer patients in early stages of disease, when cancer cells in blood are especially rare. To date, the utility of acoustic separation was proved in clinical samples from MBC patients (*n* = 3) [168].

### Comparison of methods efficiency for epithelial and mesenchymal CTCs recovery in spike-in tests

#### Recovery rates (RR) of CTCs by different methods

The choice of CTCs isolation method has a great impact on the sample positivity rate, especially when one aims at detecting mesenchymal CTCs. The method’s CTCs recovery rate (RR) is usually verified by spike-in tests. In general, a known number of cells from cancer cell lines is added into the defined volume of peripheral blood or PBMCs. Next, whole sample is processed, and spiked cells are counted and/or recovered, and the RR is usually counted as follows:

$$ {\text{Recovery rate}} = \frac{{\text{Number of recovered cells}}}{{\text{Number of spiked cells}}} \times 100\% $$

To examine which methods are the most appropriate for recovery of mesenchymal BC CTCs, we performed literature review to search for studies which have performed spike-in experiments with BC cell lines with a variety of CTCs isolation methods and retrieved the information on the mean RRs (± SD) and number of test repeats (*n*). EMT score of BC cell lines was obtained from literature [171] and used to define epithelial/mesenchymal phenotype (Supplementary Table 1). Results were compared with the recovery rate obtained by CellSearch^®^, current golden standard in CTCs isolation.

#### Recovery rates of epithelial and mesenchymal cell lines in spike-in tests

**Fig. 1:**
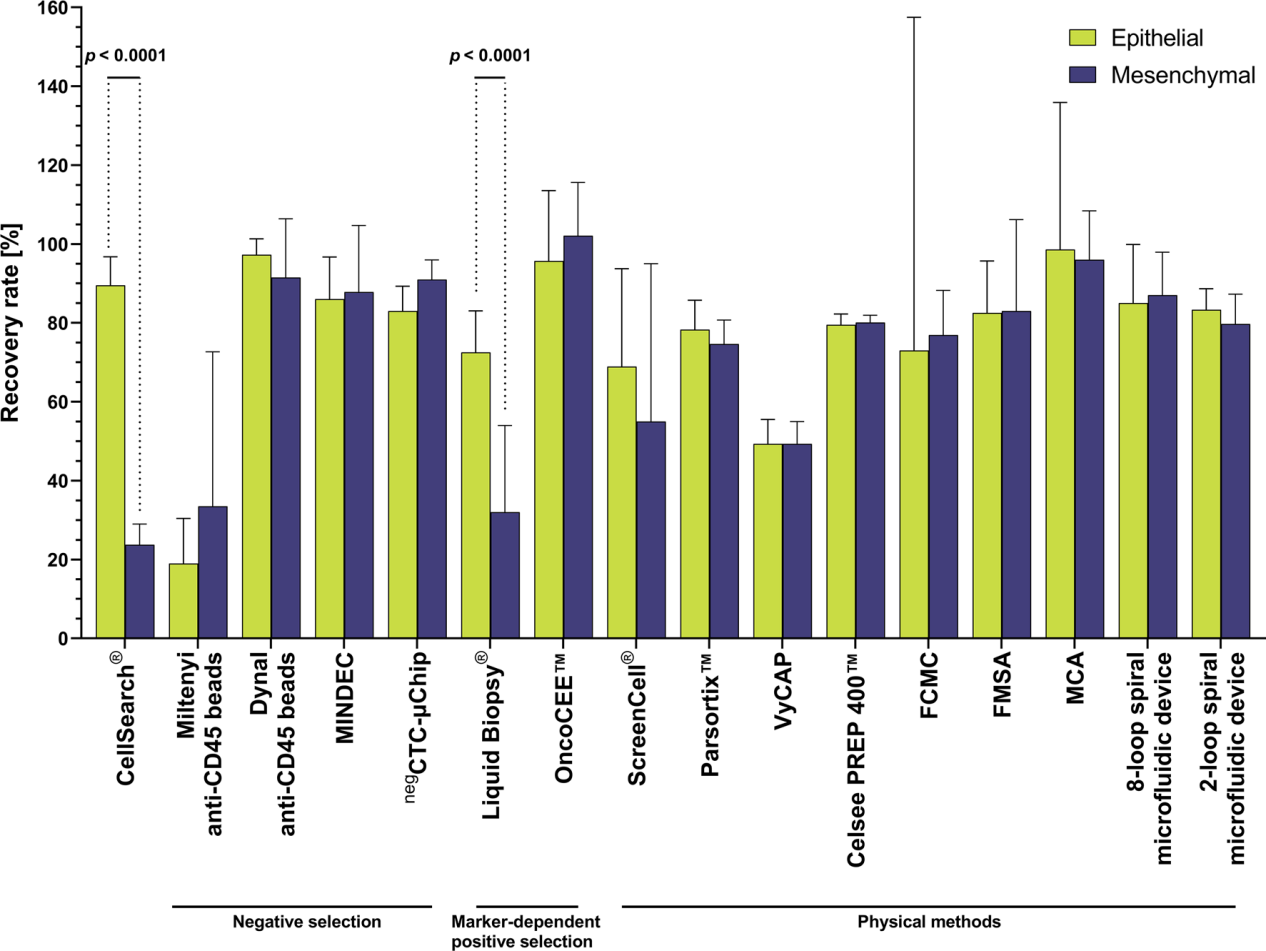
Recovery rates of epithelial (green) and mesenchymal (blue) cell lines isolated by different methods. Only methods in which both epithelial and mesenchymal BC cell lines were analyzed were included in the analysis (CellSearch^®^ [172–176], Miltenyi anti-CD45 beads [83], Dynal anti-CD45 beads [83], MINDEC [84], negCTC-µChip [78], Liquid Biopsy^®^ [98], OncoCEE™ [103], ScreenCell^®^ [133, 177], Parsortix™ [145], VyCAP [147], Celsee PREP 400™ [178], FCMC [172], FMSA [126], MCA [179], 8-loop spiral microfluidic device [149], 2-loop spiral microfluidic device [150]). Bars show means and lines shows 95% CI. Recovery rates were compared using two-way ANOVA and Sidak’s multiple comparisons test

Comparing the ability of individual methods to isolate epithelial and mesenchymal CTCs, majority of the negative selection (Dynal anti-CD45 beads, MINDEC, ^neg^CTC-Chips) and physical properties-based methods (Parsortix™, VyCAP, CelSee PREP 400™, FCMC, FMSA, MCA, spiral microfluidic devices) yielded similar RRs of spiked cell lines, regardless of their EMT phenotype (Fig. 1). The only exception is LiquidBiopsy^**®**^ system and CellSearch^®^. In fact, CellSearch^®^, representing epithelial marker-dependent positive selection, showed the highest disproportion in epithelial and mesenchymal cells recovery rates, with clear preference towards cells with epithelial phenotype (RR of 89.5% for epithelial cells vs 23.7% for mesenchymal cells, *p* < 0.0001, Fig. 1).

Looking more generally on the methods, we classified isolation approaches into groups: (1) CellSearch^®^, (2) negative selection, (3) marker-dependent positive selection (apart from CellSearch^®^), (4) physical methods. In the case of epithelial cells, negative selection (RR 71.1%) and marker-dependent-positive selection (RR 68.4%) resulted in decreased recovery in comparison to CellSearch^®^ (RR 89.5%; *p* = 0.0096, *p* = 0.001, respectively, Supplementary Fig. 1). At the same time, physical methods (RR 79.6%) ensure higher recovery than marker-dependent-positive selection (*p* = 0.037). For the mesenchymal cells isolation, CellSearch^®^ was characterized by the lowest RR (23.7%) in comparison to the physical methods (which showed the best performance, RR 74.7%, *p* < 0.0001), negative selection methods (RR 70.8%, *p* < 0.0001) and marker-dependent positive selection (RR 62.9%, *p* < 0.0001). However, individual methods within groups showed much different RRs, e.g., Miltenyi anti-CD45 beads showed much decreased RRs in comparison to the other negative selection methods in the group (Supplementary Fig. 2A and B) and LiquidBiopsy^®^ had decreased mesenchymal cells RRs in comparison to OncoCEE in the marker-dependent positive selection group (Supplementary Fig. 2B).

**Fig. 2:**
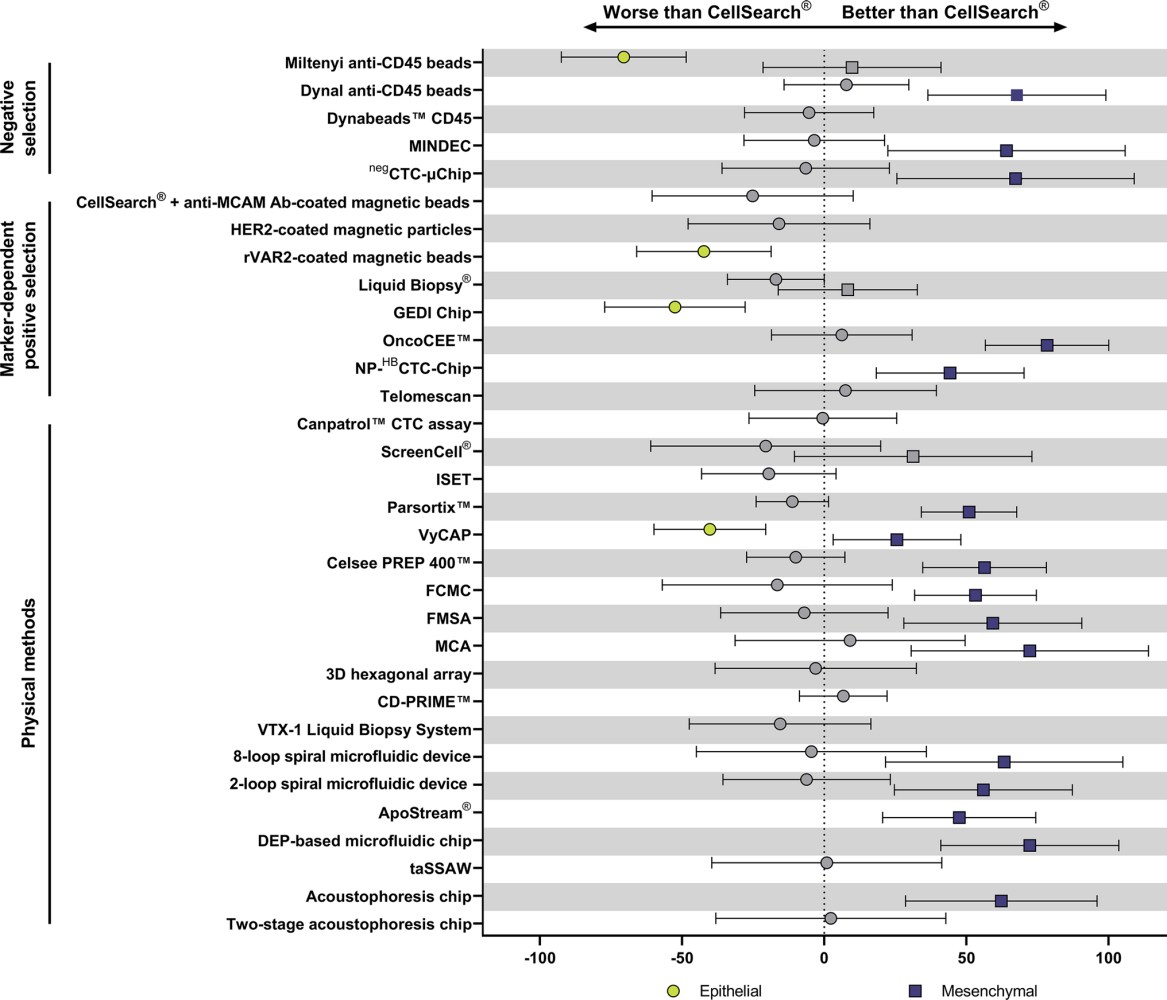
The mean differences between recovery rates of epithelial (circle) and mesenchymal (square) cell lines obtained by chosen method (Miltenyi anti-CD45 beads [83], Dynal anti-CD45 beads [83], Dynabeads™ CD45 [181], MINDEC [84], negCTC-µChip [79], CellSearch^®^ + anti-MCAM Ab-coated magnetic beads [42], HER2-coated magnetic particles [88], rVAR2-coated magnetic beads [96], Liquid Biopsy^®^ [98], GEDI Chip [101], OncoCEE™ [103], NP-^HB^CTC-Chip [99], Telomescan [109], Canpatrol™ CTC assay [182], ScreenCell^®^ [133, 177], ISET [127], Parsortix™ [145], VyCAP [147], Celsee PREP 400™ [178], FCMC [172], FMSA [126], MCA [179], 3D hexagonal array [183], CD-PRIME™ [177], VTX-1 Liquid Biopsy System [154], 8-loop spiral microfluidic device [149], 2-loop spiral microfluidic device[150], Apostream^®^ [160], DEP-based microfluidic chip [161], taSSAW [168], acoustophoresis chip [169], two-stage acoustophoresis chip [170]) and CellSearch^®^ [172–176] (showed on *Y* axis). Bars indicate 95% CI. Statistically significant results (*p* < 0.05) are shown in green (epithelial) and blue (mesenchymal), whereas non-significant are shown in grey. Recovery rates were compared using two-way ANOVA and Dunnet’s multiple comparisons test

When individual methods were compared directly with CellSearch^®^, negative selection methods, such as Dynal anti-CD45 beads, MINDEC and ^neg^CTC-µChip provided higher recovery of mesenchymal cells than CellSearch^®^, and at the same time were not inferior to CellSearch^®^ in the recovery of epithelial cells (Fig. 2). Of the negative selection methods only Miltenyi anti-CD45 beads were worse in recovery of epithelial cells. On the other hand, Miltenyi anti-CD45 enrichment allows for achieving lower contamination of cells with PBMCs (median 0.5%) in comparison to Dynal anti-CD45 beads (median 20.0%) [83]. Also methods isolating CTCs based on physical properties were generally superior to CellSearch^®^ in isolating mesenchymal CTCs, but not different in isolating epithelial CTCs (Fig. 2, e.g., Parsortix™, CelSee PREP 400™, FCMC, FMSA, MCA, spiral microfluidic devices). Though VyCAP showed decreased recovery rate of epithelial cells in comparison to CellSearch^®^, recent report performed on mouse blood with spiked human epithelial BC cancer cell line showed that VyCAP outperforms CellSearch^®^ in epithelial BC cells recovery [180]. RRs of cell lines with epithelial and mesenchymal phenotypes were not available for all of the methods, in such cases only one of the phenotypes is shown on Fig. 2. Comparison of all methods with each other is shown in the Supplementary Fig. 2.

One of the limitations of the comparison of different methods was that they come from different laboratories, which might already introduce variation in the RRs. We were able to assess lab-to-lab variation only for the CellSearch^®^ method, for other techniques there were not enough data originating from different laboratories to perform such analysis. For CellSearch^®^, variation in RRs of three different cell lines (MCF-7, MDA-MB-231, SK-BR-3) between different laboratories was not significant (Supplementary Fig. 3). However, CellSearch^®^ is a well standardized, semi-automated method which might show lower variation in comparison to methods which involve more laborious handling. We have also compared the influence of (1) the number of cells spiked-in to blood, (2) blood volumes used for the analysis, (3) the way of detecting spiked-in cells, which was either by counting pre-stained cells or staining cells during/after the enrichment procedure. It was not possible to account for all these differences at the same time due to limited number of data points. However, from the data obtained for the individual methods (where experiments with different number of spiked cells were performed in one set of conditions), we could observe that the number of spiked-in cells was often not related to differences in the RRs (Supplementary Fig. 4A). For Miltenyi anti-CD45 beads there was a decrease in the RR of cell if ≥ 100 cells were added (vs < 10 cells added) and for recovery of MDA-MB-231 with Dynal anti-CD45, where 10 spiked-in cells were more efficiently recovered than 100 cells, however the difference was not statistically significant. Analysis performed for all the methods together did not show systemic difference in the RRs (Supplementary Fig. 4B), though one can suspect that a loss of only few cells would have more significant effect on an experiment in which 10 cells were added, than on an experiment with 1000 spiked-in cells. We have not seen differences in RRs of spiked-in cells depending on the blood volume used (less than 5 mL vs equal or more than 5 mL, Supplementary Fig. 4C), however analyses with spiked-in cells are just model systems, where often significant number of cells are added into a small blood volume (e.g., 1 mL), whereas in patients’ samples more blood is processed to capture much smaller numbers of CTCs. The type of staining of the spiked-in cells was also not related to systemic differences in RRs (Supplementary Fig. 4D), however in the spike-in tests, assessment of the RRs is either based on adding pre-stained cells to blood (therefore not relaying on any external detection marker) or use optimal detection marker (highly expressed) for a given spiked-in cell line, which is difficult to identify in heterogeneous population of CTCs found in patient’s samples. Therefore, the spike-in models fail to account for the effect of CTCs detection marker, which is required for identification of CTCs in patients’ samples might be an important contributor to CTCs detection rate.

## Methods and markers for mesenchymal CTCs detection and characterization in clinical samples

Unlike in the spike-in experiments described above, where pre-stained BC cell line cells are frequently added to blood samples, CTCs in patients’ samples have to be additionally identified with specific markers, usually by immunofluorescent staining or by gene expression analysis. Therefore, CTCs positivity rate (PR) in patients’ samples is affected by (1) the type of the method of CTCs-enrichment and (2) the markers used for CTCs detection. To evaluate how CTCs-enrichment and detection marker are related to mesenchymal CTCs positivity, we have analyzed data from studies isolating/detecting CTCs from BC patients with different methods and non-epithelial detection markers. We have included classical EMT markers, like VIM, CDH2 (N-cadherin), EMT transcription factors (TWIST1, SNAIL, SLUG, ZEB1) as well as other proteins, which were shown to induce EMT or which expression was upregulated during EMT (EGFR, MCAM, MUC1, PI3K, AKT2, BIRC5, HER3, TG2, FOXC2, MAGEA3). In total, we collected the data from 31 studies which were detecting non-epithelial CTCs markers in BC patients (Supplementary Table 2). For further statistical analysis ten studies were removed due to either (1) not specifying CTCs PRs separately for non-metastatic (M0) and metastatic (M1) patients or individual markers, (2) collecting blood sample after primary tumor removal or (3) including only CTC-positive patients (during earlier testing) for the analysis, which could influence mesenchymal markers PRs. Included studies were testing groups of BC patients ranging from 9 to 446 women. Distant metastasis status of patients was extracted from the publication, analyses were done separately for non-metastatic and metastatic patients. The final list of studies and data used for statistical analysis and modelling is presented in Supplementary Table 3.

Each study was classified according to the CTCs enrichment method and CTCs marker used for CTCs detection. Regarding CTCs enrichment method, we created three groups:

1. NEG—negative selection methods (depletion of CD45-positve cells with immunomagnetic particles, one study was based on filtration of full blood),
2. PosEPI—positive epithelial selection methods (anti-EpCAM covered immunomagnetic particles, CellSearch^®^, anti-CK MACS enrichment),
3. PosMES—positive mesenchymal selection methods (AdnaTest, AdnaTest EMT-2/StemCellSelect, modified CellSearch^®^ with anti-EpCAM and MCAM immunomagnetic particles, anti-csVIM enrichment).

Next, studies were divided according to the non-epithelial marker used for detection of CTCs. For some markers only one data point was available, making statistical analysis between the markers not possible, therefore such cases were excluded from the analysis of between marker comparisons. Data used for statistical analyses in this section are given in the Supplementary Table 3.

We defined mesenchymal CTCs positivity rate as the percentage of patients in which CTCs with any of the above given marker was detected. These markers are not specific for fully mesenchymal CTCs, and can be found in CTCs with varying levels of EMT activation, which could also include intermediate EMT phenotype (with co-existing epithelial features).

### CTCs positivity rates according to the detection marker and selection method in clinical samples

**Fig. 3:**
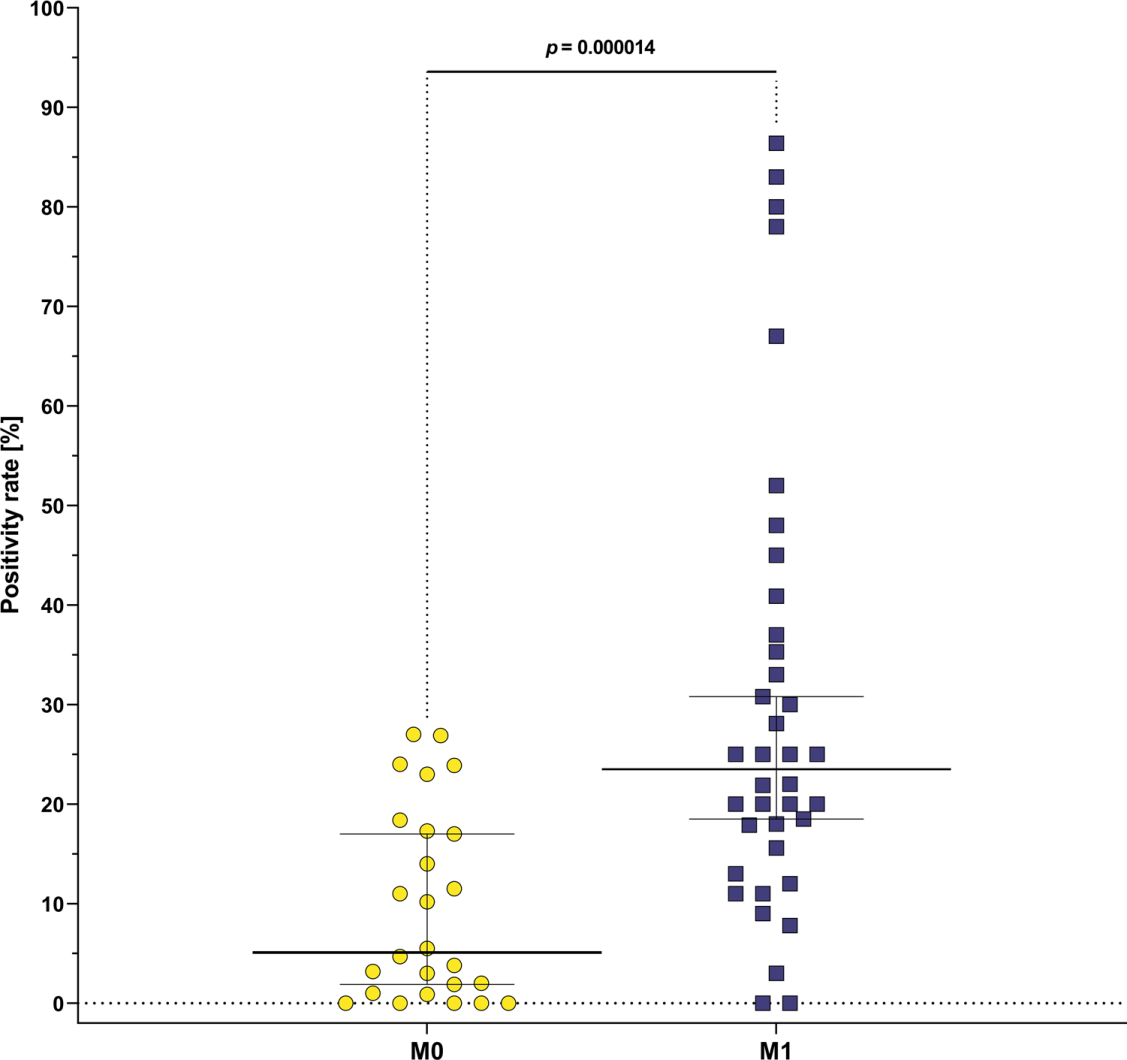
Overall mesenchymal CTCs positivity rates in M0 (yellow) [7, 43, 48, 56, 70, 87, 184–188] and M1 (blue) [12, 42, 70, 76, 77, 185–187, 189–193] patients compared using Mann–Whitney test. Bars show 95% CI and horizontal lines show medians

Overall, considering all markers and CTCs enrichment methods used, mesenchymal CTCs PRs were lower for M0 than for M1 patients (median 5% vs 23%, respectively, *p* = 0.000014, Fig. 3). None of the markers showed difference in PRs between M0 and M1 patients, though VIM showed a trend towards higher PRs in M1 (59.5%) than in M0 group (20.5%, *p* = 0.13, Supplementary Table 4A). When all EMT-TFs (TWIST1, SNAIL, SLUG, ZEB1) were grouped and analyzed as one variable, there was higher median PRs in M1 (18.2%) patients than in M0 (1%, *p* = 0.03; Supplementary Table 4A). However, in case of EMT-TFs, they were combined with different types of enrichment—in M0 patients EMT-TFs were analyzed in CTCs isolated mostly via negative enrichment (80% of the samples), whereas in M1 patients in EMT-TF were tested via negative enrichment in 40% of the cases (another 40% were analyzed by positive epithelial enrichment and 20% by positive mesenchymal enrichment).

Regarding CTCs enrichment methods, positive mesenchymal selection resulted in higher CTCs PRs in M1 (25.0%) patients than in M0 (10.2%, *p* = 0.002; Supplementary Table 4B); similarly negative selection yielded higher PRs in M1 (25%) than in M0 patients (1.4%, *p* = 0.00037; Supplementary Table 4B).

**Fig. 4:**
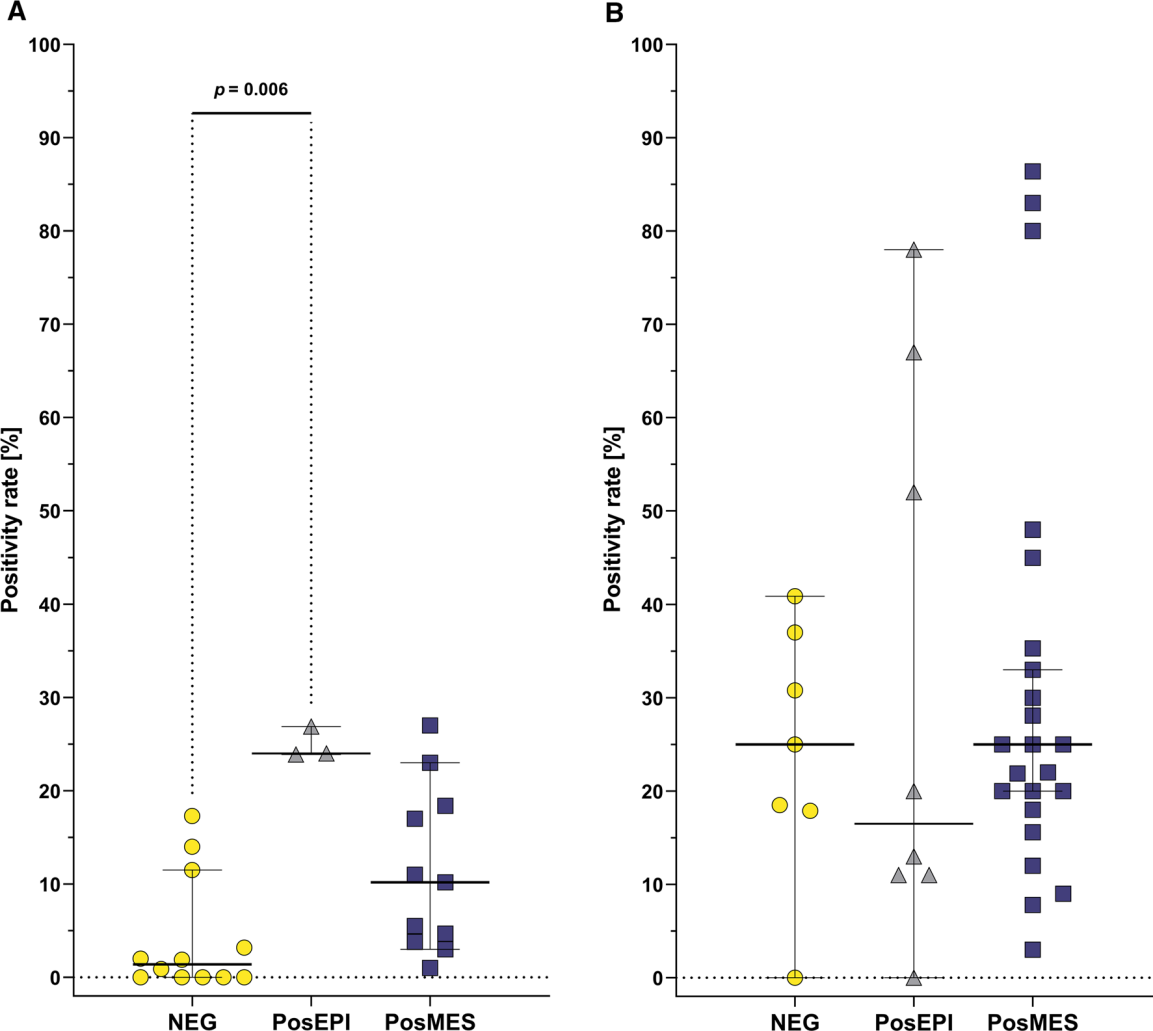
Mesenchymal CTCs positivity rates according to CTCs isolation method in M0 (**A**; NEG [7, 48, 56], PosEPI [187, 188], PosMES [43, 87, 184–186]) and M1 stage (**B**; NEG [7, 48, 77, 192, 193], PosEPI [77, 187, 191], PosMES [12, 42, 70, 76, 185, 186, 189, 191]) patients. Individual methods were compared using Kruskal–Wallis test. Bars show 95% CI and horizontal lines show medians

When M0 and M1 patients were analyzed separately, in the M0 group positive epithelial selection (24.9%) was better in CTCs isolation than negative selection (1.4%, *p* = 0.006; Fig. 4A). However, only three data points were available for positive epithelial selection and additional studies would be required for more robust interpretation of this finding. In the M1 group there was no difference between the CTCs isolation methods (Fig. 4B).

**Fig. 5:**
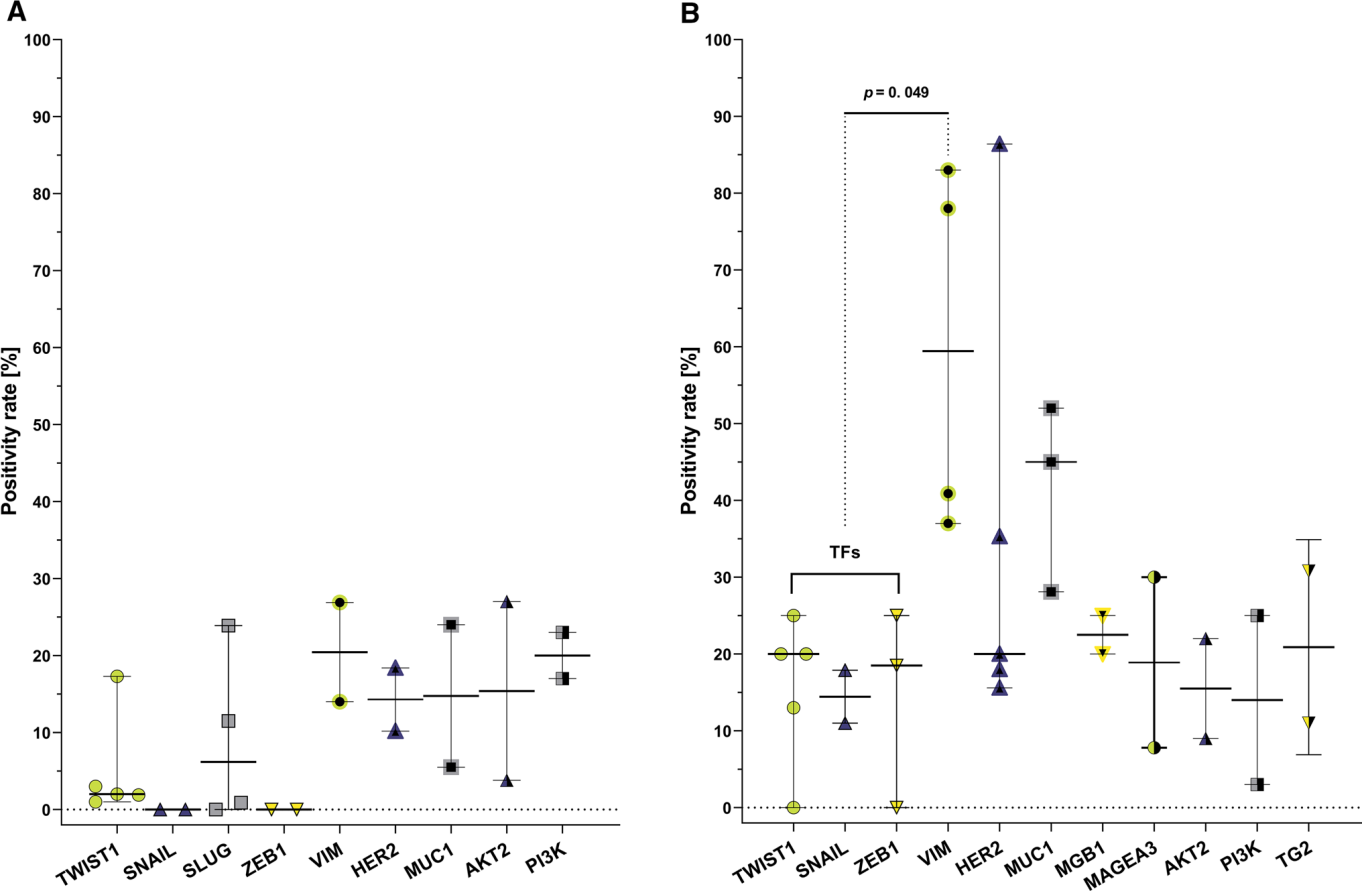
Mesenchymal CTCs positivity rates according to CTCs-detection marker group in M0 (**A**; TWIST1 [7, 48, 56, 87, 184], SNAIL [7, 56], SLUG [7, 48, 56, 188], ZEB1 [7, 56], VIM [48, 188], HER2 [185, 186], MUC1 [185, 187], AKT2 [87, 184], PI3K [87, 184]) and M1 stage (**B**; TWIST1 [70, 77, 191], SNAIL [77], ZEB1 [77, 192], VIM [12, 190, 192, 193], HER2 [76, 185, 186, 189, 191], MUC1 [185, 187, 189], MGB1 [189, 191], MAGEA3 [185, 191], AKT2 [70, 76], PI3K [70, 76], TG2 [77]) patients. Individual markers were compared using Kruskal–Wallis test, additionally EMT-TFs were grouped and compared with other markers. Bars show 95% CI and horizontal lines show medians

Considering markers used for mesenchymal CTCs detection, there were no statistically significant differences between the markers in M0 and M1 patients (Fig. 5A). However, when individual EMT-TFs (TWIST1, SNAIL, SLUG, ZEB1) were grouped, they showed decreased mesenchymal CTCs positivity rate (18.2%) in comparison to VIM (59.5%, *p* = 0.049) in the M1 patients (Fig. 5B). It has to be emphasized that limited data points were available for many of the investigated markers or positive epithelial selection, therefore, one has to be cautious while interpreting this finding. Nevertheless it still seems of interest with respect to selection of CTCs enrichment methods and markers and is thus surely worth verification in much larger dataset.

### Modelling the impact of CTCs enrichment method and detection marker on CTCs positivity rate in BC patients

Due to the fact that individual detection markers were used in combination with different CTCs-enrichment methods (negative selection, positive epithelial selection or positive mesenchymal selection), it is difficult to deduce how specific detection marker and selection method impact the efficiency of CTCs capture. Therefore, statistical modelling was additionally employed to assess the impact of individual detections markers and enrichment methods on CTCs PR.

#### Modelling the impact of CTCs enrichment method and detection marker on CTCs positivity rate in BC patients

To evaluate the suitability of various selection methods and detection markers for obtaining high CTCs PRs, the CTCs PR was analyzed as a function of selection method and detection marker by means of the partial least squares (PLS) regression modelling. To this end, CTCs PRs reported by studies enrolled in the review were considered as continuous response variable dependent on selection method and detection marker used in the study, both of which were considered as two independent discrete predictors. The number of components in the PLS model was set based on the criterion that each component involved in the model should explain more than 1% of the total response variability. The PLS regression model was then fitted to obtain the estimates of regression coefficients (*β*) for all levels of these predictors. Sigma-restricted regression model was employed with the negative selection method and SNAIL marker used as reference (as they were linked with the lowest PRs in M0 patients) to which all other selection methods and markers were compared. All selection methods and detection markers were then ranked according to their *β*-values estimates. The higher the *β*-value of a given selection method or detection marker, the more positively it contributes to the CTCs detection. Analysis and modelling is presented in Table 1.

**Table 1:** Results of the PLS regression modelling evaluating the suitability of various CTCs selection methods and detection markers for obtaining high CTCs positivity rates

Factors affecting the CTCs positivity rate	M0			M1		
	*β*	*R*	*R*_w_	*β*	*R*	*R*_w_
Marker: SNAIL	ref	–	–	ref	–	–
Selection: NEG	ref	–	–	ref	–	–
Marker: PI3K	**21.21**^a^	1	2.73	− 14.21	12	9.76
Selection: PosEPI	**18.32**^b^	2	2.24	5.30	5	5.52
Marker: AKT2	16.87	3	4.09	− 12.45	11	9.76
Marker: HER2	15.83	4	4.00	7.75	4	5.09
Marker: VIM	8.85	5	4.88	**40.90**^c^	1	1.23
Marker: MUC1	5.82	6	6.56	18.42	2	3.21
Marker: TWIST1	4.53	7	6.65	− 5.88	9	8.83
Marker: SLUG	2.57	8	7.02	–	–	–
Marker: ZEB1	− 2.35	9	9.00	− 0.11	7	7.84
Selection: PosMES	− 3.43	10	7.83	13.56	3	3.72
Marker: TG2	–	–	–	2.48	6	6.44
Marker: MGB1	–	–	–	− 4.27	8	7.88
Marker: MAGEA3	–	–	–	− 8.48	10	8.72

Presented are the estimated values of regression coefficients (*β*) for all investigated selection methods and detection markers involved in PLS regression modelling, together with resultant ranks (*R*) and weighted ranks yielded by the bootstrap validation (R_W_). Values in bold indicate statistically significant *β* estimates. Factors affecting CTCs positivity rate are listed in descending order based on their *β* estimate values in the M0 group of patients

^a^*p* < 0.05; *H*_1_: *β*_PI3K_ > *β*_SNAIL_ (in the case of M0)

^b^*p* < 0.05; *H*_1_: *β*_PosEPI_ > *β*_NEG_ (in the case of M0)

^c^*p* < 0.005; *H*_1_: *β*_VIM_ > *β*_SNAIL_ (in the case of M1)

Furthermore, estimates of *β* were then tested against their permutational distributions obtained from 50,000 permutations under two zero hypotheses (*H*_0_) saying that obtained *β* estimates are not significantly (1) higher or (2) lower compared to the reference (i.e., to the *β* coefficients of negative selection and SNAIL marker, assumed to equal zero). Statistically significant differences were assumed for *p* < 0.05.

Finally, the ranking of selection methods and detection markers based on their *β*-value estimates was validated using the bootstrap technique. The whole PLS regression-based ranking procedure was repeated 50,000 times to yield a bootstrap estimate of the distribution of ranks for all selection methods and detection markers. Selection methods and markers were then ranked according to their weighted average ranks (R_W_) which were compared to those from the initial PLS model and the whole distribution of ranks was presented in the form of a heatmap.

All statistical analyses were performed in R using the *pls* and *superheat* packages [194, 195].

#### Results

PLS regression modelling, employed to investigate the relationship between the CTCs positivity rate and different selection methods and detection markers used for CTCs detection, was performed separately for M0 and M1 stage patients and the results are summarized in Table 1.

In the case of the M0 stage patients, the PLS model explaining 72% of total response variability revealed that positive epithelial selection method has the highest and statistically significant estimate of the *β* coefficient (*β* = 18.32, *p* < 0.05 (compared to negative selection (NEG)) providing thus the strongest enhancement of the CTCs positivity rate. This indicates that when using positive epithelial selection methods for CTCs isolation, significantly higher CTCs PRs can be achieved than using negative selection. For the CTCs detection markers, PI3K had the highest *β* coefficient (*β* = 21.21, *p* < 0.05), which was significantly higher than the one of SNAIL (reference marker).

For M1 patients, PLS model, explaining 54% of the total response variability, showed that the detection marker VIM has the highest estimate of the *β* coefficient, with its value being significantly higher (*β* = 40.90, *p* < 0.005) than the one of SNAIL (reference). This indicates that VIM is a significantly better mesenchymal CTCs marker than SNAIL.

#### Validation of PLS regression results

Rankings of all investigated selection methods and detection markers yielded by the two PLS models were positively validated using the bootstrap technique (Supplementary Fig. 5) as the rankings based on weighted ranks (*R*_W_ in Table 1) were analogous to those obtained according to values of *β* estimates from the two initial models (*R* in Table 1). Even though there are some minor discrepancies apparent between rankings based on *R* and *R*_W_, these are mainly limited to swapped places between two neighbouring markers/selection methods with the general trend remaining the same. In the case of the M0 stage, the positive epithelial selection method and PI3K were found to place 1st and 2nd, in 41.7% and 35.9% of all bootstrap runs, respectively; while ZEB1 transcription factor was placed on last 10th place in 33.0% of all runs. In the case of the M1 stage, VIM marker and positive epithelial selection method were found to place 1st and 3rd in 86% and 31.9% of all bootstrap ranks, while PI3K was found on the last 12th place in 35.3% of runs.

## Summary and discussion

Interest in phenotyping CTCs populations based on epithelial-mesenchymal characteristics has increased in recent years, however CTCs capturing methods are still not always suitable for capturing mesenchymal CTCs [3, 7, 12, 43, 44, 46, 48, 51, 59, 76, 77, 87, 191, 196–198]. Addition of anti-mesenchymal and stem cells markers antibodies to classical anti-EpCAM enrichment increased the number of CTCs detected [102, 103]. Nevertheless, mesenchymal CTCs markers are often being studied in CTCs isolated via epithelial marker-based enrichment. This is possible if cells undergoing EMT still show some epithelial characteristics, but the approach is not informative if post-EMT cells are to be captured. Data show that purely mesenchymal CTCs can be observed in up to 71% of MBC and 35% of non-metastatic patients [44], and at disease progression almost all CTCs can acquire mesenchymal phenotype [3]. Therefore, if inappropriate CTCs enrichment/identification approach is used, there is a risk of high false negative CTCs detection rate [3, 12, 44]. Given that different EMT states of CTCs can provide important prognostic information it becomes more critical to appropriately design protocols for CTCs isolation and identification/characterization in clinical samples.

Data from BC cell lines spike-in experiments showed that the majority of the methods designed to enrich various EMT phenotypes of CTCs were better than CellSearch^®^ in isolating mesenchymal CTCs, with only few showing compromised epithelial CTCs recovery. None of the methods showed significantly higher RRs of epithelial cells than CellSearch^®^, justifying CellSearch^®^ being the golden standard in epithelial CTCs isolation. Methods allowing mesenchymal CTCs isolation showed rather similar performance in epithelial CTCs recovery, but differences were observed when mesenchymal CTCs were evaluated. This comparison has however several limitations—the compared methods were performed in different laboratories, often using different blood volumes, different number of cells spiked-in, and different cells staining techniques (adding pre-stained cells or staining cells after the procedure). Using pre-stained cells or applying the most optimal marker for detection of given spiked-in cell lines might result in high recovery rates, but fails to account for heterogeneity in CTCs observed in patients. Therefore performance of methods might differ between the simulated spiked-in sample and real clinical sample. This was the incentive for analyzing mesenchymal CTCs detection rate in BC samples, with a focus on the type of the method for CTCs enrichment and marker used for CTCs detection in BC patients. This analysis showed that in general mesenchymal CTCs are more frequently detected in patients with metastatic BC (M1 stage) than in patients with no distant metastases (M0). In M1 BC patients there was no significant difference in performance of the CTCs-enrichment methods (positive epithelial, positive mesenchymal or negative selection), whereas in M0 patients the positive epithelial selection resulted in the highest CTCs positivity rates (PRs), which was significantly better than negative selection (though only three studies were part of the positive epithelial selection group). This conclusion was supported by the result of PLS regression analysis. When mesenchymal CTCs markers were analyzed by simple test for statistical significance of differences (i.e., the Kruskal–Wallis H test), it has not revealed any differences in CTCs PRs depending on the marker used in the M0 patients. At the same time PLS regression showed that PI3K was associated with higher PRs than EMT-TF SNAIL, however only two data points were available for PI3K. The most commonly used markers for mesenchymal CTCs detection were EMT-TFs (TWIST1, SNAIL, SLUG, ZEB1). We did not observe differences in CTCs PRs among these markers (both in M0 and M1 patients), but when they were combined and analyzed as a single group (as EMT-TFs), they showed to perform worse than VIM in M1 patients. Also in the PLS regression model, VIM was identified as a better marker than SNAIL in M1 patients. Outcomes of the PLS regression modelling suggest that when detecting CTCs in patients’ samples, it is possible to obtain higher positivity rates than those provided by the commonly used EMT transcription factors (SNAIL, SLUG, TWIST, ZEB1) simply by selecting PI3K or VIM (in M0 or M1 patients, respectively). This conclusion seems to be supported by the fact that SLUG, TWIST and ZEB1 presented close-to-zero (positive or negative) estimates of *β* regression coefficients, which, consequently, resulted in their performance not being significantly different from that of the reference marker (SNAIL). On the other hand, PI3K and VIM were found to present significantly higher values of *β* estimates, indicating that their usage in CTCs detection can be significantly more beneficial in terms of high CTCs positivity rates compared to reference (SNAIL) and, plausibly, also by other EMT-TFs analyzed in this study. This conclusion would, however, benefit from further verification in larger study setting, as the hereby presented analysis still suffers from several shortcomings, mainly low number of data points in several groups and possible collinearity between compared markers. These might have resulted in decreased statistical power as well as in our inability to include interactional terms in PLS model, thus forcing us to analyze slightly simpler model involving the main effects only. Additional data from future studies would be needed to make statistical analysis and modelling more accurate and, ultimately, to verify our conclusion. Moreover, modelling performed in the group of M1 patients explains only about 50% of the CTCs positivity rates, showing that there are other factors underlining variability in CTCs numbers in patients, not accounted by the model. This could be due to methodological differences in the analysis of genes expression or proteins level as well as dissimilarity in the study groups.

Comparing data from spiked-in BC cells lines with patients’ samples, it seems that higher mesenchymal CTCs detection rate in patients could be obtained if methods not relying only on epithelial markers (e.g., selected physical methods, negative selection methods or multimarker-based enrichment like OncoCEE™) would be more frequently used. Currently however, physical enrichment methods (which were frequently tested on spiked-in epithelial and mesenchymal BC cell lines) are not commonly applied for phenotypic characterization of CTCs from clinical samples, and we could not perform statistical analysis for this type of CTCs enrichment methods for patients’ samples. On the other hand, analysis of CTCs detection rate in patients’ samples revealed that positive epithelial selection is better than negative selection in M0 patients, but in M1 patients no differences between the methods were observed. We therefore presume, that spike-in models might not easily be translated to patients’ samples as the issue of marker used for CTCs detection in patients’ samples is not adequately addressed in spike-in models. Regarding optimal marker for mesenchymal CTCs detection more data is needed to unequivocally indicate the best candidate. Transcriptome analysis of CTCs on a single cell level will hopefully allow to identify novel, more specific markers to be used in patients’ samples. Recent guidelines delineate that definition of mesenchymal or EMT-undergoing cells cannot rely on limited set of, especially pleiotropic factors, such as EMT-TFs, which can orchestrate different molecular programs [199]. As heterogeneous expression of mesenchymal/EMT markers is observed in CTCs, multi-marker approach (staining with a few different mesenchymal-related markers) is recommended to increase sensitivity. This has to be balanced against marker specificity (inclusion of negative markers in CTCs staining), as mesenchymal/EMT markers (e.g., EMT-TFs, VIM) can be expressed in blood of healthy controls [7, 46, 48, 77, 196] and their application as sole markers of mesenchymal CTCs, without additional control genes or correction for background expression level is not recommended. This seems especially crucial as data from patients’ samples reveal that mesenchymal CTCs are associated with disease progression and their targeting might be a strategy to lower metastases formation [75, 200].

## Supplementary Information

Below is the link to the electronic supplementary material.

Supplementary file1 (RAR 275 KB)

Supplementary file2 (RAR 24627 KB)

## Data Availability

All data are available as Online Recourses.

## Abbreviations

AKT2: RAC-beta serine/threonine-protein kinase
AURKA: Aurora kinase A
BC: Breast cancer
BIRC5: Baculoviral IAP Repeat Containing 5/surviving
CAR: Coxsackievirus–adenovirus receptor
csVIM: Cell-surface vimentin
CTCs: Circulating tumor cells
DEP: Dielectrophoresis
DFS: Disease-free survival
EMT: Epithelial-mesenchymal transition
EMT-TFs: EMT-associated transcription factors
FDA: Food and Drug Administration
GEDI: Geometrically enhanced differential immunocapture
*gfp*: Green fluorescent protein gene
HER2: Human epidermal growth factor receptor 2
HER3: Human epidermal growth factor receptor 3
hTERT: Human telomerase
ISET: Isolation by size of epithelial tumor cells
MACS: Magnetic cell separation system
MAGEA3: MAGE Family Member A3
MBC: Metastatic breast cancer
MCAM: Melanoma cell adhesion molecule
MET: Mesenchymal-epithelial transition
MGB1: Mammaglobin 1
MINDEC: Multi-marker immunomagnetic negative depletion enrichment of CTCs
MUC1: Mucin 1
NEG: Negative selection methods
NSCL: Non-small cell lung carcinoma
ofCS: Oncofetal chondroitin sulfate
OS: Overall survival
PFS: Progression-free survival
PLS-3: Plastin 3
PLS: Partial least squares
PosEPI: Positive epithelial selection methods
PosMES: Positive mesenchymal selection methods
PR: Positivity rate
RNA-ISH: RNA in situ hybridization
RR: Recovery rate
rVAR2: Recombinant glycosaminoglycan-binding malaria protein VAR2CSA
R_W_: Weighted average ranks
taSSAW: Tilted-angle standing surface acoustic wave
TG2: Transglutaminase 2
